# Patients’ migration for orthopaedic intensive rehabilitation among Italian Regions

**DOI:** 10.1093/eurpub/ckac131.532

**Published:** 2022-10-25

**Authors:** G Guarducci, A Urbani, S Carbone, F Moirano, G Messina, N Nante

**Affiliations:** 1 Post Graduate School of Public Health, University of Siena, Siena, Italy; 2 General Directorate for Health Planning, Ministry of Health, Rome, Italy; 3 Fucina Sanità, Cuneo, Italy; 4 Department of Molecular and Developmental Medicine, University of Siena, Siena, Italy

## Abstract

**Background:**

Interregional patients’ migration, according to Italian Law, can be considered an expression of the (inviolable?) right to health and freedom of choice regarding place of care. It contributing, albeit perversely, to guaranteeing equity in the Italian National Health Service allowing citizens to overcome territorial inequalities in the distribution of healthcare services. The aim of our study was to analyze fulfilment of needs for orthopaedic intensive rehabilitation hospital services on site and interregional patients’ migration trends.

**Methods:**

We conducted an observational cross sectional study on Hospital Discharge Cards provided by the Ministry of Health, upon specific request, from 2011 to 2019. The study of interregional patients’ migration, for orthopaedic intensive rehabilitation, relative to single Italian regions was carried out from data of Residents, Attractions and Escapes, which were graphically developed through Gandy’s Nomogram. Trend analysis (Cuzick’s Test) was performed through STATA. Were considered statistically significant at level of 95% (p < 0.05).

**Results:**

In our studied period, Gandy’s Nomogram showed that only Piedmont, Lombardy, A.P. of Trento, E. Romagna, Umbria and Abruzzo had a good public hospital planning for orthopaedic intensive rehabilitation. Attractions increased significantly for Lombardy, A.P. of Trento, Veneto and Basilicata, while they decreased significantly for A.P. of Bolzano, Veneto, F.V. Giulia, Abruzzo, Calabria and Sicily. Escapes increased significantly for Veneto, F.V. Giulia, E. Romagna, Tuscany, Molise, Puglia and Basilicata, while they decreased significantly for Piedmont, Aosta Valley, A.P. of Trento, Umbria, Abruzzo and Sicily.

**Conclusions:**

Only six regions (4 in the North, 1 in the Centre and 1 in the South) satisfied care needs of their Residents, with an Attractions minus Escapes positive balance. Only A.P. of Trento appears to have been able to reduce Escapes and increase Attractions at the same time.

**Key messages:**

• Studying patients’ migration by type of health benefit makes it possible to identify specific situations of lack of supply.

• Patients’ migration is an indirect Index of a region’s health policy.

